# Evaluation of the cost-effectiveness of the treatment of uncomplicated severe acute malnutrition by lady health workers as compared to an outpatient therapeutic feeding programme in Sindh Province, Pakistan

**DOI:** 10.1186/s12889-018-6382-9

**Published:** 2019-01-17

**Authors:** Eleanor Rogers, Saul Guerrero, Deepak Kumar, Sajid Soofi, Shahid Fazal, Karen Martínez, Jose Luis Alvarez Morán, Chloe Puett

**Affiliations:** 1 Action Against Hunger UK, 161-163 Greenwich High Road, London, SE10 0JA UK; 2 Action Against Hunger USA, One Whitehall St, New York, NY 10004 USA; 3 Action Against Hunger Pakistan, 3rd Floor, 65 West Executive Heights, Fazal E Haq Road, Blue Area, Islamabad, Pakistan; 4 Aga Khan University, Stadium Road, P. O. Box 3500, Karachi, 74800 Pakistan

**Keywords:** Cost-effectiveness, Severe Acute Malnutrition (SAM), Lady Health Workers (LHWs), Community-based Management of Acute Malnutrition (CMAM), Pakistan

## Abstract

**Background:**

Due to the limited evidence of the cost-effectiveness of Community Health Workers (CHW) delivering treatment for severe acute malnutrition (SAM), there is a need to better understand the costs incurred by both implementing institutions and beneficiary households. This study assessed the costs and cost-effectiveness of treatment for cases of SAM without complications delivered by government-employed Lady Health Workers (LHWs) and complemented with non-governmental organisation (NGO) delivered outpatient facility-based care compared with NGO delivered outpatient facility-based care only alongside a two-arm randomised controlled trial conducted in Sindh Province, Pakistan.

**Methods:**

An activity-based cost model was used, employing a societal perspective to include costs incurred by beneficiaries and the wider community. Costs were estimated through accounting records, interviews and informal group discussions. Cost-effectiveness was assessed for each arm relative to no intervention, and incrementally between the two interventions, providing information on both absolute and relative costs and effects.

**Results:**

The cost per child recovered in outpatient facility-based care was similar to LHW-delivered care, at 363 USD and 382 USD respectively. An additional 146 USD was spent per additional child recovered by outpatient facilities compared to LHWs. Results of sensitivity analyses indicated considerable uncertainty in which strategy was most cost-effective due to small differences in cost and recovery rates between arms. The cost to the beneficiary household of outpatient facility-based care was double that of LHW-delivered care.

**Conclusions:**

Outpatient facility-based care was found to be slightly more cost-effective compared to LHW-delivered care, despite the potential for cost-effectiveness of CHWs managing SAM being demonstrated in other settings. The similarity of cost-effectiveness outcomes between the two models resulted in uncertainty as to which strategy was the most cost-effective. Similarity of costs and effectiveness between models suggests that whether it is appropriate to engage LHWs in substituting or complementing outpatient facilities may depend on population needs, including coverage and accessibility of existing services, rather than be purely a consideration of cost. Future research should assess the cost-effectiveness of LHW-delivered care when delivered solely by the government.

**Trial registration:**

NCT03043352, ClinicalTrials.gov. Retrospectively registered.

## Background

Over 16 million children under-five globally currently suffer from Severe Acute Malnutrition (SAM), a condition which increases a child’s risk of death more than eleven-fold [1, 2]. Treatment for uncomplicated SAM is now commonly provided as outpatient care with weekly visits to a nurse, complemented by Ready-to-Use Therapeutic food (RUTF) rations provided in the home by the carer. Those suffering from medical complications receive care in an inpatient facility before graduating to the outpatient component. Community Health Workers (CHWs) inform the community about the availability of services and screen for cases in the community using the Mid-Upper Arm Circumference (MUAC) measurements, referring cases for care [3]. This approach, known as Community-based Management of Acute Malnutrition (CMAM) is now used in over 70 countries worldwide, with care provided as part of a comprehensive primary health care package [1].

The CMAM model delivers high quality care, and crucially it has been found to be a cost-effective intervention in multiple contexts. In Ethiopia, the cost per child treated was 135 USD compared to 285 USD for inpatient care [4]. The cost per disability-adjusted life year averted was 42 USD in Malawi [5] and 53 USD in Zambia [6]. However, the most significant economic advantage of the model over inpatient care is the reduced financial burden to the beneficiary household of accessing care. In Ethiopia, the cost to the household was 6 USD (rounded) per child treated compared with 21 USD (rounded) for inpatient care [4]. In spite of the cost-effectiveness for both provider and beneficiary, a key limitation of the current CMAM model is its ability to achieve high levels of coverage. This has in part been attributed to the high opportunity costs of accessing care on a weekly basis, related to lost income and the cost of transport to the health centre which can in some contexts be far from the beneficiary home [7–9].

In recent years, an alternative delivery model for SAM has emerged, building on the success of the integrated Community Case Management (iCCM) approach [10]. iCCM uses trained CHWs to deliver a package of interventions, most commonly for diarrhoea, pneumonia and malaria, in their own community using simplified treatment protocols. This model has proven to be effective in reducing barriers to access, by reducing case severity due to earlier case finding and management [10]. Evidence on the cost-effectiveness of this approach remains limited, although a multi-country analysis found that due to high fixed costs it can be a costly intervention if service utilisation is low [11]. The treatment of SAM without medical complications has been trialled as part of such a package of interventions in Bangladesh and Ethiopia, showing that CHWs are able to provide an acceptable level of care [12, 13]. In Bangladesh, the high quality of care that CHWs achieved in managing cases of SAM [14, 15] did not result in lower quality on other routine tasks [16] and was accomplished despite common systemic challenges in extending care to populations with limited access to services in low resource settings [17]. Moreover, data from Bangladesh shows that this can be a cost-effective approach at 26 USD per DALY averted, including costs to both institutions and beneficiaries. Perhaps the most significant advantage to the model, demonstrated in Bangladesh, was the greatly reduced costs to beneficiary households relative to inpatient care [13].

Due to the limited evidence of the cost-effectiveness of CHW-delivered treatment for SAM, there is a need to better understand the costs incurred by both implementing institutions and beneficiary households. This would provide additional evidence to potential implementers on whether it is a feasible alternative to the current CMAM model. This study assessed the costs and cost-effectiveness of treatment for uncomplicated SAM delivered by Lady Health Workers (LHWs) employed within the national health system and complemented with non-governmental organisation (NGO) delivered outpatient facility-based care, (hereafter referred to as LHW-delivered care) compared with existing outpatient health facilities operated by an NGO (hereafter referred to as outpatient facility-based care) in Sindh Province, Pakistan. Costs were estimated for implementing institutions and beneficiaries, and cost-effectiveness was assessed per child recovered from SAM.

## Methods

### Description of the intervention

This study partnered with the LHW Programme in Pakistan, a well-established national CHW program set up in 1994 as part of the National Programme for Family Planning and Primary Health care. Under the government programme, LHWs undergo 6 months of training to provide both preventive and treatment services [18]. Between March 2015 and April 2016 Action Against Hunger, an international NGO working in Pakistan since 2005, partnered with Aga Khan University (AKU) to test the feasibility of delivering treatment for SAM without complications through LHWs in a two-armed cluster randomised controlled trial (trial registration number: NCT03043352).

In the intervention arm 72 LHWs, who were part of the existing government programme, screened for SAM, treated cases of SAM without medical complications and referred any complicated cases to the health centre. Children aged 6–59 months meeting the eligibility criteria and for whom parental consent was provided were enrolled and provided medical and nutrition treatment complemented with counselling on nutrition and Infant and Young Child Feeding (IYCF) practices in their home, with follow up until recovery. Children eligible for inpatient care were referred to the nearest hospital. Three pre-existing outpatient facilities remained open in the intervention arm as it would not have been ethical to remove an existing care provider for the duration of the study. In the control arm, 72 LHWs screened for SAM referring all cases to the outpatient health facility for treatment, having been trained to do so for this study. Action Against Hunger staff provided outpatient treatment as per national CMAM protocol and children were followed up by the LHW at household level with counselling on nutrition and IYCF. As in the intervention arm, any child suffering from medical complications was referred to the nearest hospital for inpatient care. Training for LHWs on the CMAM protocol was provided by Action Against Hunger, who also supervised, monitored and provided logistical support, including the provision of RUTF. The trial examined the impact of integrating the screening and treatment of SAM into the LHW’s existing role, in terms of effectiveness, coverage, quality of care and cost effectiveness.

Service delivery was assessed in terms of standard treatment outcomes, and rate of recovery from SAM was the primary study outcome. A total of 430 children were admitted into the intervention arm and 399 into the control arm. Table 1 shows trial outcomes for both arms. A ‘defaulter’ is classified as a child that is absent from treatment for three consecutive weeks and a non-responder is a child that fails to respond to treatment after 16 weeks, including referral to inpatient care where a treatable cause cannot be found. Results from the effectiveness study have been documented elsewhere [19].

**Table 1:** Cohort outcomes

Outcome	Intervention		Control	
	Number	Percent	Number	Percent
Recovered	323	76.0	326	83.0
Defaulted	16	3.8	10	2.5
Dead	1	0.2	2	0.5
Non-responder	85	20.0	55	14.0
Total discharged	425	100.0	393	100.0

### Analytical strategy

This cost-effectiveness analysis compared the costs incurred during community mobilisation and treatment by LHWs and in three outpatient centres in the intervention arm with those incurred in the control arm in outpatient facility-based care. Standard methods were employed for cost-effectiveness analysis within Action against Hunger [20], including adopting a societal perspective to include costs incurred by beneficiaries and the wider community. Costs were estimated through key informant interviews and informal group discussions, and determined to belong to the intervention or the control arm respectively. This analysis accounts for the one-year implementation period of the project, from April 2015 to April 2016. Cost-effectiveness ratios (CER) were calculated per child recovered from SAM.

### Data collection

All costs were estimated via review of internal documents, including programme reports and Action Against Hunger accounting records. As government and AKU budget documents were not available, cost information was obtained via key informant interview and developed using an ingredients approach to cost estimation [21]. Semi-structured interviews were conducted (*n* = 55) with LHWs, supervisors and outpatient facility based staff as well as with management, finance, logistics and monitoring staff, to discuss their involvement in and time allocated to the intervention. Five informal group discussions were conducted with beneficiary households and community members in each arm (*n* = 10 total) to estimate time spent and out of pocket costs incurred when accessing care.

### Costing assumptions

Cost estimates in this study exclude research activity costs in order to estimate costs of standard programme implementation. AKU staff conducted some screening and referral of cases in the control arm during the month of August, and provided beneficiaries with counselling related to SAM at the household level in both arms. As these activities affect enrolment and may impact nutrition outcomes in the study areas, these costs have been included in the analysis. The cost of capital items was amortized using standard Tables (3 years for computers, 5 years for other equipment) and discounted at a rate of 3%. One year of useful life for these items was included in the analysis. Costs were not adjusted for inflation as the duration of implementation was one calendar year.

### Data analysis

The activity-based costing methodology involves identifying staff time allocation to specific programme activities and to each arm of the intervention. This information was gathered during interviews and informal group discussions. All staff were consulted to develop a list of key cost centres to which costs could be allocated. Five main cost centres were identified: treatment, supervision and monitoring, training, support, and household costs. Costs were assigned according to time allocation proportions derived from interviews, and based on direct utilisation, where possible. A detailed description of each cost centre and the associated costs and data sources are outlined in Table 2.

**Table 2:** Costing and time allocation source information per activity

Cost centre	Description	Data sources
Treatment	Salaries: LHWs, Field Supervisors, outpatient and inpatient staff. Logistics (rent, utilities, storage), transport (vehicles, fuel, maintenance), RUTF supply, transport and storage and programme supplies.	Review of NGO accounting data, time allocation interviews with government, NGO field and management and AKU staff. Government costs estimated through interviews with management staff. Community time and missed labour costs estimated through time allocation interviews with key informants from community and cross checked with NGO and government staff.
Supervision & Monitoring	Salaries: Field Supervisors, outpatient Staff, NGO management, technical staff. Transport (care hire).	Review of NGO accounting data, ‘off budget’ costs for government staff and AKU estimated through key informant interviews. Time allocation interviews with government and NGO field and management staff.
Training	Location, transport, trainer and materials.	Review of NGO accounting data, ‘off budget’ costs estimated through interviews with government and Action Against Hunger staff. Time allocation interviews with field and management staff.
Support	Salaries: Logistics, finance, HR and guards. Equipment (computers, printers, cameras), office rent and utilities and rent of LHW health house.	Review of NGO accounting data, ‘off budget’ costs estimated through interviews with government and AKU staff. Time allocation interviews with field and management staff. Interviews with community leaders to estimate facilities used at community level and triangulated with data from interviews from Action Against Hunger and AKU.
Household	Opportunity costs of accessing treatment and money spent accessing services.	Informal Group Discussions with beneficiary households on time allocated to accessing treatment, financial costs and lost income.

Costs and effects were modelled using TreeAge Pro 2016 software. Both intervention and control arms were analysed in separate models, comparing costs and outcomes to a “do nothing” alternative, assuming zero costs and no recovery of children from SAM; these separate models isolated the costs and effects of the intervention and control arms, to assess how they performed independently using average cost effectiveness ratios (ACER). An incremental cost effectiveness analysis was conducted to assess the additional cost per child recovered from SAM in the control arm relative to the intervention arm using an incremental cost effectiveness ratio (ICER). In this way, this study compares results of each arm relative to no intervention, and incremental to an existing intervention, providing information on both absolute and relative cost-effectiveness [21].

Sensitivity analyses were conducted to determine whether plausible variations in programme costs and outcomes would have resulted in a significant change in the final CER for each intervention and the conclusion as to which program was more cost-effective. Univariate sensitivity analyses were conducted by varying one parameter at a time across a range of plausible values. Multivariate probabilistic sensitivity analyses were conducted to assess joint variation in all parameters, using 100,000 iterations per model. Gamma distributions were used to characterize cost parameters and beta distributions were used for recovery rates.

For univariate sensitivity analyses, plausible variation was defined as follows. In both arms maximum and minimum cost was defined as +/− 25% of the base case. Published historical data was used to determine a plausible range of CMAM recovery rates, with a maximum rate of 95% and a minimum of 60% assumed [9]. The same range was used for each arm, as there is currently inadequate data to inform a standard performance range of recovery rates specifically for CHWs managing cases of SAM.

## Results

### Costs

Total programme costs in the base case were 123,497 USD for LHW delivered care (the intervention arm) and 118,198 USD for outpatient facility-based care (the control arm). Table 3 presents in detail the costs allocated to each arm.

**Table 3:** Input costs for the intervention and control arms

	Intervention		Control	
	USD	% of total costs	USD	% of total costs
Personnel	53,542	43.4	48,937	41.4
LHWs (salaried and volunteer incentives)	10,330	8.4	6572	5.6
Field staff (community mobilisation, supervisors, medical and data collectors)	23,853	14.3	27,554	17.9
Management staff	13,811	16.2	6311	10.8
Support staff (logistics, finance, administrative)	5547	4.5	8500	7.2
Programme costs	36,525	29.6	21,316	18.0
Office and programme materials	13,384	10.8	3269	2.8
RUTF (supply)	19,410	15.7	18,011	15.2
Training costs (trainer, location, supplies)	3731	3.0	36	0.0
Logistics	26,049	21.1	35,480	30.0
Rent and utilities (&storage)	8006	6.5	13,807	11.7
Transport (car rental, maintenance & fuel)	18,043	14.6	21,673	18.3
Community contributions:	7381	6.0	12,465	10.5
Costs to households	4123	3.3	9207	7.8
Opportunity costs for community leaders	2766	2.2	2766	2.3
Community-level rent	492	0.4	492	0.4
Total	123,497	100.0	118,198	100.0
Cost to Institutions	92,343	74.8	84,488	71.5
Cost to Government	23,773	19.2	21,245	18.0
Cost to Communities	7381	6.0	12,465	10.5

The largest input cost category was for personnel which was similar in both arms (53,542 USD in the intervention and 48,937 USD in the control), although in the intervention arm, the LHW associated costs were higher (10,330 USD compared to 6572 USD in the control arm).

Programme costs were higher in the intervention arm, including the cost of training the LHWs to provide treatment, at 3731 USD, although no costs of training the outpatient facility-based medical staff in either arm were included in this analysis for comparison. The costs of transport, rent and utilities were high in both arms, although less in the intervention compared to the control. The costs of the monitoring team from AKU were included as they conducted some case finding and counselling in each arm, which included car rental costs of 7752 USD for each arm, significantly inflating the transport costs.

One of the most significant cost differences was the cost to the households which was double in the control arm compared to the intervention arm at 9207 USD and 4123 USD respectively, despite a similar number of children being treated. This cost covers money spent on transportation to the health facility or LHW health house, any additional funds spent on food for beneficiaries during the treatment period, and foregone income. As health facilities were further from beneficiary homes than the LHW health house, the majority of the increased cost associated with receiving outpatient facility-based care is the cost of transport.

### Activity-based costs

**Fig. 1:**
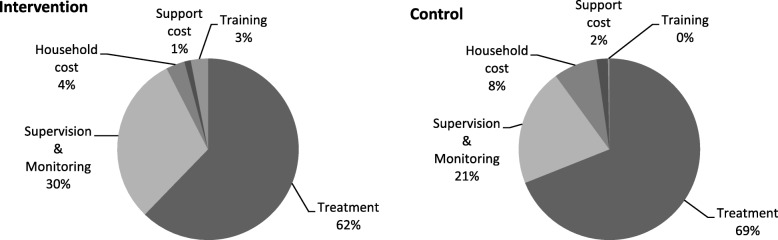
The proportion of costs per activity for each arm

When costs were assigned to one of the five cost centres identified for this programme: treatment, supervision and monitoring, training, support or household costs, the proportion of costs assigned to each activity was similar between arms (see Fig. 1). The most costly activity in both arms was treatment, amounting to 60–70% of the total costs, and containing costs for LHWs, supervisors, medical staff and management salaries, logistics and RUTF. Supervision and monitoring was the next most costly activity in both arms, although it comprised a greater proportion of total costs in the intervention arm. This was due to Action Against Hunger project management staff time spending 70% of their time on this activity in the intervention arm compared to 30% in the control arm.

The cost to the household of receiving outpatient facility-based care was double that of LHW delivered care.

### Cost-effectiveness

Base case cost-effectiveness results are shown in Table 4. The average costs were 291 USD per child treated and 382 USD per child recovered for LHW delivered care. Average costs were similar for outpatient facility-based care, at 301 USD per child treated and 363 USD per child recovered.

**Table 4:** Base case cost-effectiveness results

Outcome	Intervention	Control
Total cost (USD)	123,497	118,198
# children in programme	425	393
Recovery rate	76.00%	82.95%
Number of children recovered	323	326
Cost per child treated (USD)	291	301
Cost per child recovered (USD)	382	363
Incremental costs (USD)		10.18
Incremental effectiveness		6.95%
ICER (USD)		146

*USD* US Dollar, *ICER* incremental cost-effectiveness ratio

The incremental analysis found that the cost savings per child treated in the control arm relative to the intervention arm were 10 USD, and the control arm achieved a recovery rate that was nearly 7% higher relative to the intervention arm. The resulting ICER indicates that an additional 146 USD was spent per additional child recovered in the control arm compared to the intervention arm.

### Sensitivity analysis

Parameter values and ranges used in the sensitivity analyses are presented in Table 5.

**Table 5:** Model parameter values and ranges

Parameter	Base case	Worst case	Best case	Source
Recovery rate, intervention	76.00%	60.0%	95.0%	Base – cohort study Worst/Best – historical programme performance [9]
Recovery rate, control	82.95%	60.0%	95.0%	
Cost per child, intervention	291	363	218	Base – average cost per child Worst/Best – +/− 25% of base case
Cost per child, control	301	376	226	

### Univariate sensitivity analysis

Results from the univariate analysis were similar across all models (analysis not shown). In the intervention arm, both the cost and recovery rate variables demonstrated similar levels of uncertainty. Varying only the cost parameter resulted in a change in the base case ACER of 287 to 478 USD in the intervention arm and from 272 to 453 USD in the control arm. Varying the recovery rate from maximum to minimum values resulted in a change in the ACER from 306 to 484 USD in the intervention arm compared to the base case of 382 USD, and from 317 to 501 USD in the control arm, compared to the base case of 363 USD. These results indicate that even given plausible levels of variation, the cost per child recovered would not decrease substantially to fall within the same range as other published cost effectiveness analyses for the management of SAM (range: 152–193 USD (figures adjusted for inflation, presented in 2016 USD)).

In the incremental model comparing the control and intervention arms, the cost variables showed higher levels of uncertainty than outcome variables. Varying the cost per child in each arm resulted in a change of over 2000 USD in the ICER; when varying costs in the control arm the ICER varied from − 935 to 1228 USD, in the intervention arm the resulting change in ICER was from − 899 to 1192 USD. Varying the recovery rate in each arm resulted in smaller changes in the ICER; in the case of the intervention arm recovery rate parameter the resulting change was from − 308 to 947 USD, and in the control arm the change was from − 140 to 679 USD. Negative values demonstrate areas of domination and resulting uncertainty in which strategy was considered more cost-effective in all scenarios. Findings from the univariate sensitivity analysis for the incremental model indicate that the cost to recover an additional child from SAM in the control area compared to the intervention area either could result in cost savings or be as high as 1228 USD. This is more than 8 times the base case ICER (146 USD) and suggests that these results are subject to considerable uncertainty.

### Probabilistic sensitivity analysis

**Fig. 2:**
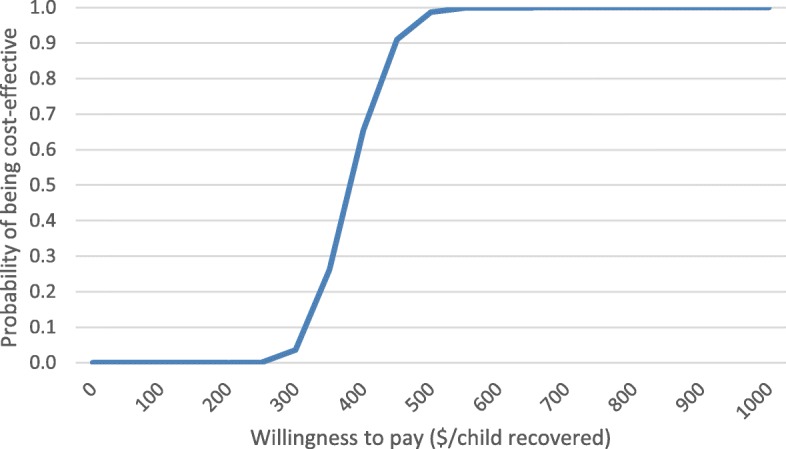
Acceptability curve for LHW delivered care in the intervention arm

Results from probabilistic sensitivity analyses are presented as acceptability curves for the intervention, control arm and the incremental analysis. The acceptability curve for the intervention arm in Fig. 2 shows the probability that the intervention would be cost-effective was 25, 50 and 75% at a willingness to pay of 347, 380 and 419 USD, respectively. The 95% confidence interval of the CER in the intervention arm was 293 to 484 USD.

**Fig. 3:**
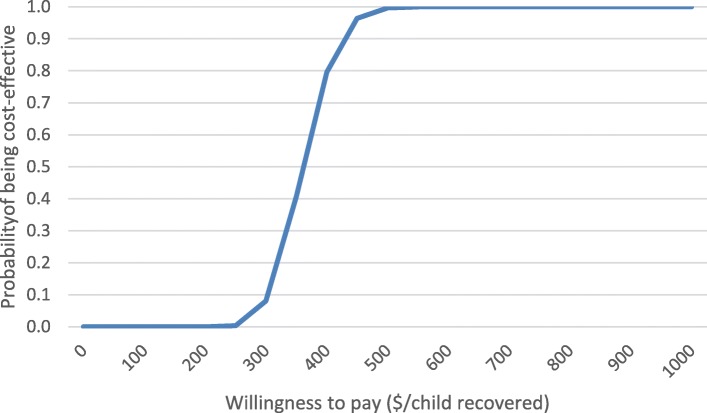
Acceptability curve for outpatient facility-based care in the control arm

Figure 3 presents the acceptability curve for the control arm, indicating that outpatient facility-based care was 25% likely to be cost-effective if society were willing to pay 326 USD per case of SAM recovered, 50% likely to be cost-effective at 362 USD per case recovered, and 75% likely to be cost-effective at a willingness to pay of 394 USD per case recovered. The 95% confidence interval for the CER in the control arm was 278 to 459 USD.

**Fig. 4:**
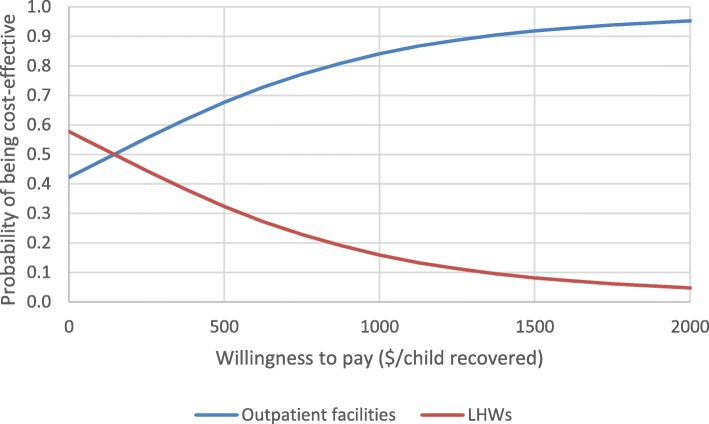
Acceptability curves for outpatient facility-based care relative to LHW-delivered care

Figure 4 presents results from the probabilistic sensitivity analysis for the incremental model. These results show that at most levels of willingness to pay, the outpatient facilities in the control arm were likely to be considered cost-effective in this setting relative to community-based management by LHWs. For example, at a willingness to pay of 500 USD, there was a nearly 70% probability that the control arm would be cost-effective compared to the intervention arm. As willingness to pay increased, so did the likelihood that the control arm would be considered more cost-effective since it was more expensive than care by LHWs in the intervention arm (in terms of cost per child treated) but also more effective. The 95% confidence interval for the ICER was − 2022 to 2665 USD, with the negative values again indicating areas of dominance.

The range of results from the univariate and multivariate probabilistic sensitivity analyses showed that accounting for plausible variation, the two interventions under analysis had costs per child recovered that were above the range of existing estimates in the published literature, and which fell between 270 and 500 USD. Results from the incremental probabilistic sensitivity analysis indicated that in most scenarios the outpatient facility-based care was considered cost-effective relative to LHW-delivered care.

## Discussion

In this setting, the existing model of outpatient facility-based care to manage cases of severe acute malnutrition (SAM) without medical complications was more cost-effective than care delivered by LHWs in communities. While these findings were subject to considerable uncertainty given the relatively small differences in costs and effectiveness between the two models, this does indicate that decentralizing management of SAM to community health workers may not always represent a reliable and effective source of cost savings despite promising findings in other settings [13].

The point estimates and ranges of the cost per child treated and recovered by LHWs at 291 USD and 382 USD were notably higher than previous results from other published analyses. In Bangladesh, where CHWs treated cases of SAM without medical complications in the community, the cost per child treated was less at 170 USD and the cost per child recovered was 186 USD (figures adjusted for inflation, presented in 2016 USD) [13]. Compared to the unit cost results in the Bangladesh study, the present study’s estimates were more than double the cost per child recovered. Additionally, the control arm in the present study which reflects a similar intervention strategy to other outpatient facility-based programmes previously assessed in Ethiopia, Malawi and Zambia [4–6], also incurred higher costs per child treated and per child recovered compared to these earlier studies, indicating there were potentially similar factors in both arms of the current study inflating the costs relative to these other previous study settings.

While direct comparisons can be fraught with challenges based on differences in methods and country-level cost structures, [22] some basic comparisons can be made to explain these disparities. First, strong effectiveness is a key factor in achieving cost-effectiveness; in the outpatient facility-based care provided in the control arm of the present study, the recovery rate was lower (at 83%) than that found in similar studies (ranging from 91 to 94%). Additionally, the cohort size was smaller. As fixed costs remain the same regardless of the number of children enrolled, this apparent low quality of care would have increased the cost per child recovered.

These two factors also contributed to an increased cost per child in the LHW-delivered care compared to similar studies. The recovery rate in the Bangladesh study was 92% compared to 76% in the LHW arm in the present study. Although the total programme costs were similar in the two settings, in Bangladesh more children were treated (*n* = 724) than in the present study (*n* = 425) [13]. In this study only 56% of admissions to the intervention arm were included in the cohort, with the remainder deemed to have been defined falsely as SAM due to inaccurate anthropometry; suggesting LHWs faced challenges in consistently performing correct measurements. In the intervention arm, two additional factors were identified as contributing to increased costs. In the intervention arm of this study, three outpatient facilities continued to provide care for cases of SAM without complications, which increased the cost in this arm. Additionally, some of the cost difference between these studies could be due to methodological differences, specifically the allocation of support costs via accounting records in the present analysis, which would have predisposed the present analysis to accounting for a more exhaustive array of costs than might have been included in a purely ingredients-based approach such as that taken in the Bangladesh study.

Although a linked study showed that LHWs in this setting were able to correctly identify SAM, and that the majority provided appropriate care, it also revealed that not all were able to do so consistently [23]. This limited the intervention’s effectiveness and therefore its cost-effectiveness. Quality of care in this setting was linked to a number of factors; first, LHWs were reportedly overburdened as they continued their routine tasks alongside polio campaigns, which left little time for SAM treatment [19]. Second, LHWs did not receive a salary top-up for delivering SAM treatment which combined with the increased workload, limited their motivation to treat cases comprehensively in a context where it is customary for all new health packages to provide an additional stipend. Third, ways of working between the government and Action Against Hunger were not clearly agreed prior to the start of implementation due to a change in government staff between the planning and implementation phases. This resulted in the Lady Health Worker Supervisors (LHS), whose role was to supervise and monitor LHW performance, not participating for the first half of the project. This likely would have not only limited the LHW’s quality of work but also limited their motivation if their supervisors were not in support of the intervention. Fourth, only 67 of the original 72 LHWs included in the study were continually active throughout the study. This was because some trained LHWs were not local residents, while some married and subsequently left the study area, both common challenges for LHW programmes [24]. Conversely in Bangladesh, CHWs delivered a high quality of care [14, 15] without it affecting the quality of their other tasks [16]. Field level monitoring in Bangladesh incurred very low costs yet allowed CHWs to ask questions and have confidence in their case management skills, [13] a factor missing from the present study. The challenges faced by this study reflect the importance of having clear agreement between implementers as well as contributing to existing evidence from Sindh and elsewhere that CHWs require sufficient supervision and monitoring, a manageable workload and appropriate financial reimbursement [24–29].

Crucially, this study found that there was little variation between the cost of recovering a child through LHW delivered care with outpatient facility-based care compared to outpatient facility-based care only in this context, at 382 USD and 363 USD respectively. As a result, based on the findings of this study, the choice of whether it is appropriate to have CHWs function as a substitute or complement to outpatient health facilities may depend on the needs of the population, including coverage and accessibility of existing services, rather than be purely a consideration of cost.

### Costs

One of the most significant findings of this study was the lower cost to the beneficiary household of receiving care from an LHW closer to their home than the facility, removing a significant barrier to access faced by the traditional CMAM model [7, 8, 30]. Although LHW delivered care was decentralised only to the village level, rather than the household, it resulted in an important reduction in opportunity costs due to travel relative to attending a facility. This finding supports existing evidence that bringing services closer to the community through CHWs can remove a key barrier to access and potentially increase service coverage [13, 15].

The most costly activity in both arms of the study was treatment, accounting for at least 60% of total costs. The cost of NGO-delivered services in each arm were high. If the government were to manage LHW delivered care, these costs would likely be reduced; future research should be conducted to assess the costs and effectiveness achieved in SAM management in such an environment. However the proportion of costs spent on supervision and monitoring in this study were low, accounting for only 30% compared to 51% in Bangladesh [13]. Combined with the challenges in maintaining high quality of care in this study, a greater focus on monitoring and supervision may be required in similar future programmes. High start-up costs are common in iCCM programmes and tend to decrease marginally over time, [11] although CHWs require strong monitoring and supervision structures, and these should be included in programme budgets [11]. Outpatient facility-based staff in both arms were previously trained and experienced in delivering care prior to the study, therefore the costs to train these staff were not included in this analysis. These costs would need to be accounted for in future programmes.

This study had two key limitations. First the intervention arm combined LHW delivered care with outpatient facility-based care so it was not possible to assess the outcomes and costs of the two delivery methods independently. Second, although the LHWs were part of an existing government programme, they received significant support from an NGO. Additionally, no official data on implementation costs was shared by the government or other implementing partners so these costs were estimated through interviews. Further research on the actual costs of LHW-delivered care that would be incurred by the government would be of value to guide future implementation of this service delivery model having LHWs manage cases of SAM at community level.

## Conclusion

The cost-effectiveness of LHW delivered care complemented by outpatient facility-based care in this setting was found to be poor, although the potential for cost-effectiveness of management of SAM by CHWs has been demonstrated in other settings. The challenges faced by the LHW model in delivering high quality services demonstrate that community-level health workers require strong supervision and monitoring, a manageable workload and context-specific, appropriate remuneration to improve effectiveness and cost-effectiveness. Additionally, it is important to achieve consensus among actors in local political systems when integrating nutrition into iCCM strategies implemented by government-employed CHWs. The cost-effectiveness of LHW-delivered care and the NGO-run outpatient facility-based model were found to be similar. This suggests that whether it is appropriate to engage LHWs in substituting or complementing outpatient facilities may depend on population needs, including coverage and accessibility of existing services, rather than be purely a consideration of cost. Future research should be conducted to assess the cost-effectiveness of LHW delivered care when implemented and supported solely by the government.

## Abbreviations

AKU: Aga Khan University
CER: Cost-Effectiveness Ratio
CHWs: Community Health Workers
CMAM: Community management of Acute Malnutrition
iCCM: Integrated Community Case Management
IYCF: Infant and Young Child Feeding
LHS: Lady Health Supervisors
LHWs: Lady Health Workers
NGO: Non-governmental Organisation
RUTF: Ready to Use Therapeutic Food
SAM: Severe Acute Malnutrition
